# The doll therapy as a first line treatment for behavioral and psychologic symptoms of dementia in nursing homes residents: a randomized, controlled study

**DOI:** 10.1186/s12877-021-02496-0

**Published:** 2021-10-12

**Authors:** Francesca Santagata, Massimiliano Massaia, Patrizia D’Amelio

**Affiliations:** 1 Department of Medical Science, Geriatric and Bone Diseases Unit, University of Turin, corso Dogliotti 14, 10126 Torino, Italy; 2 Department of Medicine, Service of Geriatric Medicine & Geriatric Rehabilitation, University of Lausanne Hospital (CHUV), Lausanne, Switzerland

**Keywords:** Dementia, Behavioral and psychologic symptoms of dementia, Nursing home, Doll therapy, Delirium, Non-pharmacological approach

## Abstract

**Background:**

Patients living with dementia are severely affected by the development of behavioral and psychologic symptoms (BPSD) which represent a burden for patients and caregivers. The use of psychotropic drugs in the control of BPSD is widely diffused, however the use of a first line non-pharmacologic approach is highly recommended.

Here we evaluate the effect of doll therapy (DT) in the management of BPSD, on the reduction of caregiver burden and delirium incidence in nursing home residents by a randomized controlled trial.

**Methods:**

We enrolled fifty-two nursing homes residents living with dementia and BPSD. Subjects were randomized to DT (26) or standard treatment (ST, 26), we measured BPSD, caregiver burden and delirium with standard clinical scales at baseline, after 45 and 90 days.

In order to evaluate the presence of BPSD we used Neuropsychiatric Inventory (NPI) scale and the A.Di.CO scale, the caregiver burden was measured by the Greutzner scale and delirium by the Confusion Assessment Method (CAM) scale.

**Results:**

DT was more effective in reducing agitation and aggressiveness as respect to ST. Moreover DT globally reduced the presence of BPSD as dysphoria, wandering and apathy. We observed a significant reduction of the professional caregiver burden and the incidence of delirium was significantly reduced in subjects treated with DT.

**Conclusions:**

We show that DT is more effective that ST in the control of BSPD in patients affected by moderate to severe dementia. Moreover we suggest that DT may effective in reducing the incidence of delirium.

**Trial registration:**

Retrospectively registered in ClinicalTrials.gov the 10th June 2, 2021 trial registration number NCT04920591.

## Introduction

During the clinical course of dementia, the appearance of behavioural and psychologic symptoms (BPSD) [1–3] severely worsen the burden of the disease. More than 80% of patients living with dementia will experience the development of BPSD, which often will cause institutionalization. BPSD include agitation, elation, wandering, depression, delusions and hallucinations [4, 5], these symptoms are difficult to manage with standard pharmacologic approach and represent a serious problem both for families and for professional caregivers in nursing homes [6, 7]. The presence of BPSD often leads to the use of multiple psychotropic drugs, as a consequence patients are exposed to severe adverse events with scarce therapeutic effect [4, 8]. Psychotropic drugs increases mortality [9] and reduce patients’ physical [10, 11] and cognitive performances [12]. De-prescribing has been proposed in order to reduce severe adverse events within a multicomponent intervention, showing improved health outcomes in old patients affected by cognitive impairment and BPSD [13]. Together with de-prescribing, several guidelines recommend the use of non-pharmacologic interventions as first line treatment for BPSD [14–16].

Amongst non-pharmacologic intervention, doll therapy (DT) has been proposed as an useful tool to reduce BPSD in patients affected by moderate to severe dementia, mostly in nursing homes [17]. Previous studies suggested that DT is useful in reducing agitation, psychotropic drug administration and in increasing patients’ quality of life [17–19]. However, despite some interesting results, published studies are mainly cohort, case-control and observational or exploratory studies [17, 20, 21].

Although the mechanism of action of DT is not fully explained, the attachment theory [21–23] has been evoked in order to explain its efficacy in the control of BPSD [21, 24]. Attachment behaviour is the tendency of every person to seek for protection and physical closeness when feeling vulnerable, this tendency persist during the whole life [25], and is particularly important in patients affected by dementia. During the course of the disease, patients become more vulnerable and some of the BPSD as wandering, dysphoria, anxiety, agitation and even aggressiveness might be interpreted as attachment requests. In this condition the doll, perceived as a translational object [19, 21, 24], catalyze patients’ attention and, hence, may reduce the attachment requests [26]. The observation of patients affected by dementia interacting with the doll shows that they treat the doll as a real baby needing care and hence they might replace their attachment request with caregiving behaviours. Frequently patients interact with the doll taking care of her needs, reassuring and lulling her. Following this theory we postulated that DT may also reduce the incidence of delirium catalyzing patients’ attention.

Despite the theoretic premises and some interesting experimental data, we are in need of controlled clinical trials to support the clinical efficacy of DT in the control of BPSD, in the reduction of caregiver burden and in the reduction of delirium.

The present study evaluate, with a randomized controlled approach, the efficacy of DT as respect to standard clinical approach in the management of BPSD, in the reduction of caregiver burden and in the reduction of delirium incidence in patients affected by moderate to severe dementia living in nursing homes.

## Methods

### Study design

This is a randomized controlled trial with two parallel arms, here we assess the effect of DT compared with Standard clinical Treatment (ST) on BPSD and on the caregiver burden in persons with dementia residents in two Italian nursing homes. The Consolidated Standards of Reporting Trials (CONSORT) guidelines for non-pharmacologic treatments has been applied [27].

### Ethical considerations and consent to participate

The Ethical Committee “Comitato Etico Interaziendale AO Città della Salute e della Scienza di Torino” approved the study (Ref. no. CE il 04/10/2018 protocol number 0098548). Written informed consent was obtained from all the participants, in patients with impaired capacity to consent a proxy consent was obtained.

### Participants

Patients residents in two Italian nursing homes (“Casa di Riposo Borsetti Sella Facenda in Mosso Biella (BI) and the “Residenza per anziani Don Giuseppe Eandi” in Lagnagsco (CN)) were enrolled in the study between the 1 January 2019 and 31 October 2019 according with the following inclusion/exclusion criteria.

#### Inclusion criteria

Age ≥ 65 years; diagnosis of dementia moderate to severe Clinical Dementia Rating scale (CDR) ≥2; presence of agitation and/or aggressiveness defined as A.Di.Co score ≥ 2; manual and visual abilities sufficient to interact with the doll.

#### Exclusion criteria

Age < 65 years; patients/relative refuse to participate; mild forms of dementia (CDR < 2); contraindication for DT; life expectancy lower than 3 months; negative interaction with the doll.

The experience of mournful events related to parental experience is considered a contraindication for the DT [28].

Patients were randomly assigned to DT or ST, the randomization was carried on computer-generated tables by the principal investigator; the patients received a consecutive number after enrolment and were subsequently allocated to randomization list, as described by Kim and Shin [29].

### Intervention

**Fig. 1:**
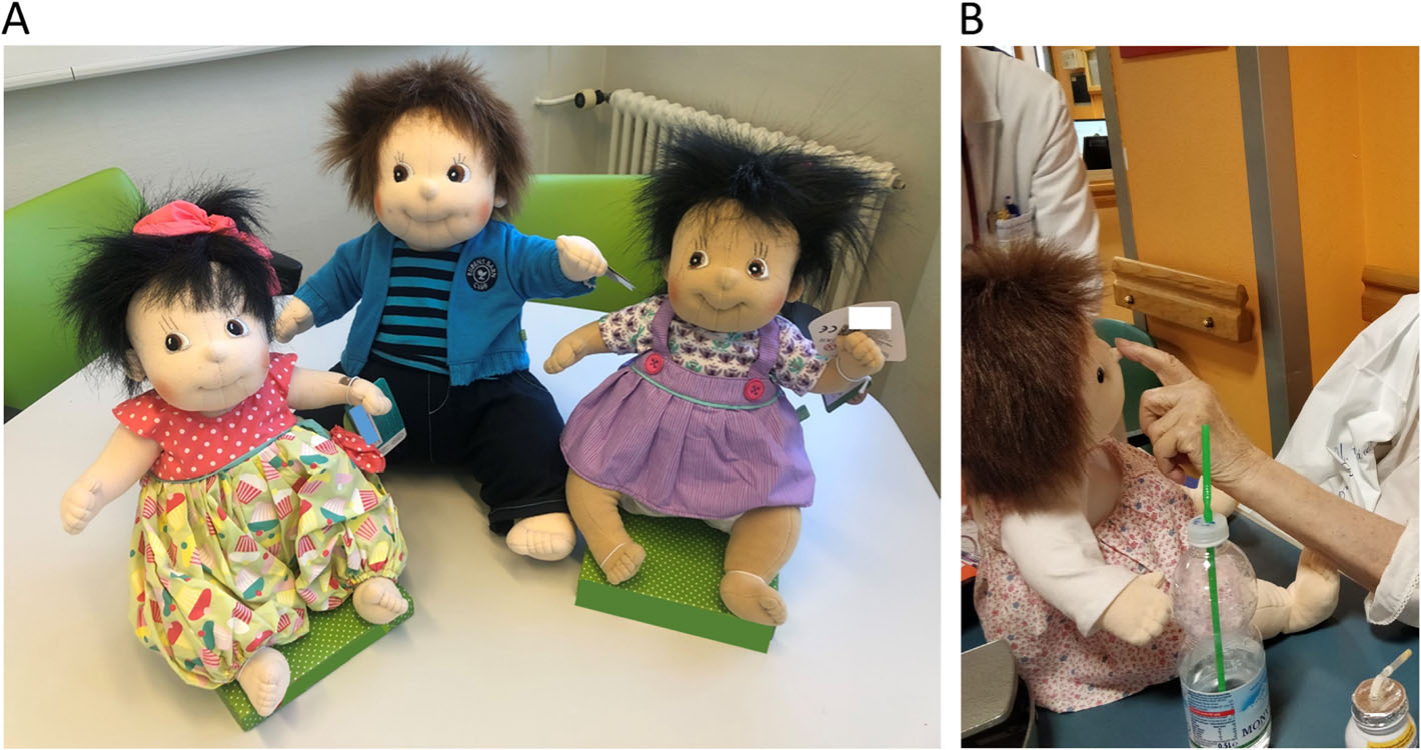
Empathy dolls. The pictures show the dolls used in the study (panel **A**), interaction between patient and doll (panel **B**)

The doll used in the study is the “empathy doll” (Fig. 1), nurses responsible for doll administration received detailed information on the aim of using DT and on the study procedure. The intervention has been fully described in our previous study [30]. The investigators informed professional and family caregivers on the mean and efficacy of DT in patients affected by dementia and BPSD, the caregiver were allowed to ask questions and received detailed answers, the concern about possible infantilization of the patients has been clearly addressed and discussed. At the end of the discussion an informative brochure on BPSD in dementia and on the role of DT was given to the caregivers, as described in our previous study [30].

The patients’ interaction with the doll was evaluated with standard methods using the Engagement Observation Rating Tool for Doll Therapy, this tool is derived from the Observational Measurement of Engagement (OME) [31] and is described in details in [32]. Patients’ interaction with doll was observed for 7 days prior to randomization and rated on the first and the last day of the week. Patients with positive attitude towards the doll were included in the study and randomized to DT or standard treatment (ST). Patients with negative interaction (refuse the doll, become agitated and or aggressive) or neutral attitude (ignore the doll) were excluded from the study.

In DT group the doll were administered two times a day for 2 hours in the morning, 2 hours in the afternoon and pro re nata (PRN) in case of agitation, aggressiveness and /or wandering. The administration of DT was the first choice in case of agitation, aggressiveness and /or wandering; if the symptoms persist, the use of pharmacologic treatment was allowed and noted. The patient can freely interact with the doll and if he/she refuses the doll, the caregiver would not insist, this type of intervention has been described in details in [30].

In the control group, the caring physician freely chose the kind of pharmacological intervention, according with standard clinical care (ST). DT and controls were comparable for antipsychotic baseline treatment (Table 1).

**Table 1:** Antipsychotic treatment at baseline and after 90 days of follow-up according to randomization. Percentage and confidence of intervals (CI) are shown

	DT (26)		ST (26)	
	*baseline (26)*	*90 days (26)*	*baseline (26)*	*90 days (26)*
**Quetiapine (%)**	27 (14–46)	26 (15–43)	27 (14–46)	32 (19–47)
**Haloperidol (%)**	23 (11–42)	9 (3–23)	19 (9–38)	26 (15–42)
**Promazin (%)**	8 (1.3–24)	18 (8–34)	12 (4–29)	5 (1–17)
**Trazodon (%)**	15 (6–34)	26 (15–43)	12 (4–29)	18 (9–33)
**Benzodiazepine (%)**	8 (1.4–24)	6 (1–19)	8 (1.4–24)	5 (1–17)
**More than 1 drug (%)**	19 (9–38)	15 (6–30)	23 (11–42)	13 (6–27)
**No drugs (%)**	0 (0–13)	0 (0–13)	0 (0–13)	0 (0–13)

### Outcomes

Primary outcomes were the reduction of BPSD and the reduction of professional caregiver burden.

Secondary outcome was the reduction of delirium.

### Analyzed variables

Presence of BPSD was evaluated with the Neuropsychiatric Inventory (NPI) scale [33] and with the A.Di.CO scale to specifically evaluate agitation and aggressiveness [30]. The A.Di.Co scale is a scale derived from the DISCO scale [34, 35], it evaluates the presence of BPSD using 10 items dived in clusters, the presence of moderate to severe agitation is scored 2.

The NPI scale was administered in a semi-structured interview setting with a close professional caregiver according with standard clinical practice described in [36]. The use of NPI allow us to evaluate the presence of several BPSD and to rate their frequency and severity. NPI evaluates different BPSD as described in [36, 37] and namely delusions, apathy, hallucinations, disinhibition, agitation/aggression, irritability, depression/dysphoria, aberrant motor behaviour, anxiety, night time behaviour disturbances, euphoria, and appetite and eating abnormalities. Each item receive a score on a 4-point scale for the frequency (0 = never, 1 = less than once a week, 2 = at least once a week, 3 = more than one a week, but less than once a day, 4 = every day). The severity of the symptom is evaluated on a 3-point scale ranging from 1 to 3 (1 = mild, 2 = moderate, 3 = severe (requires pharmacologic treatment). The distress of the caregiver regarding the behaviour is evaluated by a score ranging from 0 to 5 (0 = no stress, 1 = minimal, 2 = Mild, 3 = Moderate, 4 = Severe, 5 = very severe). The NPI total score ranges from 0 to 144 [36].

In order to specifically evaluate the professional caregiver burden the Gruetzner scale [38] was used, the presence of delirium was evaluated by the use of the Confusion Assessment Method (CAM) scale [39]. Cognition and functional status were evaluated by the Short Portable Mental Questionnaire (SPMQ) [40], the Activity of Daily Living (ADL) scale and the Instrumental Activity of Daily Living (IADL) score [41] respectively.

Age and gender were also recorded. Data analyses were blinded as respect to patients’ treatment.

**Fig. 2:**
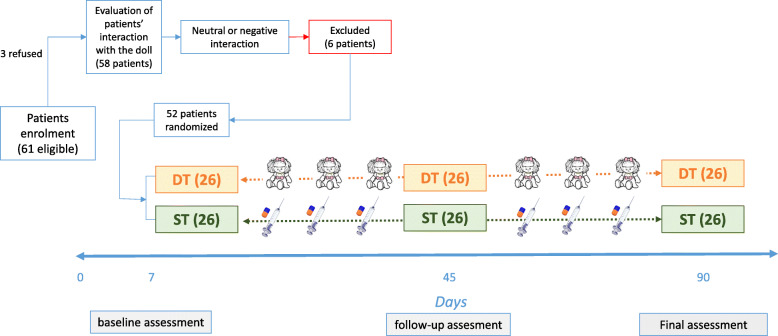
Study flow chart. The diagram shows the study design with the number of patients at each visit

Variables of interest were collected at baseline, after 45 and 90 days of intervention (Fig. 2).

### Statistical analyses

As no previous study measured the efficacy of DT in using A.Di.CO and NPI, in the period of the study drawing, the power analysis was conducted using an estimated large effect size (f = 0.40), an alpha level of 0.05, and a power of 0.8. A sample size of 52 is necessary (26 each group) for primary outcomes [42].

All the analyzed variables were tested for normality by the kurtosis test and they were all normally distributed. Patients randomized to DT were compared to patients randomized to ST by one-way ANOVA for continuous variables and by χ2 test for gender. The effect of DT was evaluated per protocol using the two-way ANOVA for repeated measurements for continuous variables and by χ2 test for trends for the incidence of delirium.

SPSS 25.0 were used for the statistical analyses and *p* < 0.05 was considered statistically significant. Graphs were drawn using GraphPad 8.0 for Windows.

## Results

Sixty-one residents in nursing homes were eligible to the study, of those 52 were recruited. Three residents refused to participate whereas six (9.6%) have a neutral or negative interaction with the doll, general characteristics of patients included in the study versus patients excluded were similar (data not shown).

The DT group did not significantly differs from patients in ST group for age, gender, cognitive performance, level of independence, presence of delirium, presence of BPSD; the only variable that significantly differs in the two groups was the professional caregiver burden measured by the Greutzner scale, which was significantly higher in DT group (Table 2). In order to correct for possible effects of baseline differences on the follow-up a Sidak’s multiple comparisons test was run-out, there were no significant difference in any of the analysed variables (data not shown).

**Table 2:** General characteristics of patients according with treatment group. Mean ± SE are shown, *p* values were calculated by one-way ANOVA and by χ2 test for gender

	DT (26)	ST (26)	***p*** value
**Age (years)**	87 ± 7	86 ± 6	0.160
**A.Di.Co****(score)**	1.3 ± 0.7	0.92 ± 0.7	0.281
**NPI (score)**	48.3 ± 12	48.2 ± 13	0.124
**Greutzner (score)**	44.3 ± 11. 6	36 ± 9.1	**0.04**
**CAM (score)**	4.0 ± 0.5	3.2 ± 0.5	0.194
**ADL (score)**	3.2 ± 0.6	3.3 ± 0.8	0.309
**IADL (score)**	1.95 ± 0.2	2 ± 0	0.312
**SPMQ (score)**	1.5 ± 0.7	1.6 ± 0.5	0.967
**CDR (score)**	2.5 ± 0.8	2.6 ± 0.5	0.270
**Women (%)**	80%	86%	0.512
**Men (%)**	20%	14%	

There were no dropouts during the study and the DT was well accepted during the whole study by patients and caregivers.

### DT is effective in reducing BPSD and caregiver burden

**Fig. 3:**
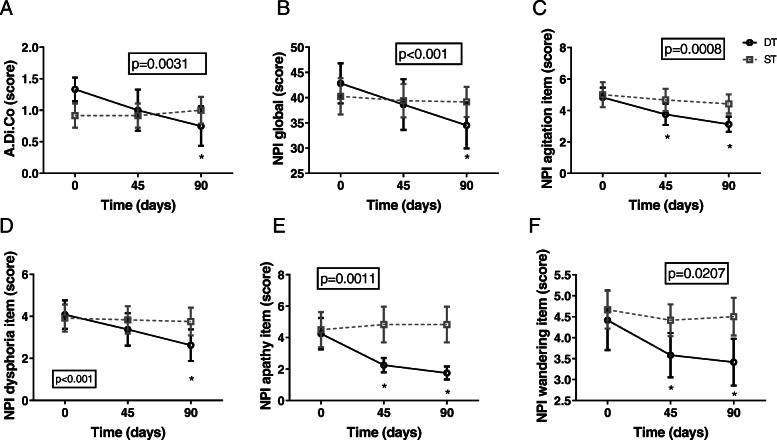
Doll therapy is effective in the control of BPSD. Effect of DT versus ST on the control of BPSD measures by A.Di.CO (panel **A**), NPI global score (Panel **B**), NPI score for agitation (panel **C**), NPI score for dysphoria (panel **D**), NPI score for apathy (panel **E**), NPI score for wandering (panel **F**). Results of two-way ANOVA for repeated measurements are shown in the box, significant differences versus baseline are indicated by the star (*)

DT was more effective in reducing agitation and aggressiveness as respect to ST, in particular we observed a significant reduction in the A.Di.Co score (Fig. 3 A) and in the items of NPI rating agitation (Fig. 3C). Moreover DT globally reduced the presence of BPSD as shown by the reduction of NPI global score (Fig. 3B), in particular we observed a significant amelioration of dysphoria (Fig. 3D), wandering (Fig. 3F) and apathy (Fig. 3E).

Eight patients out of 26 (30.7%) needed DT administration for appearance of agitation and aggressiveness. The DT was administered PRN 32 times and was effective in calming the patients 28 times (87.5%), in 4 occasion psychotropic drugs were needed to control BPSD.

We observed no significant difference in the kind of chronic antipsychotic treatment administration at baseline and after 90 days of follow-up in DT and ST groups (Table 1), nor in the dose used (data not shown).

**Fig. 4:**
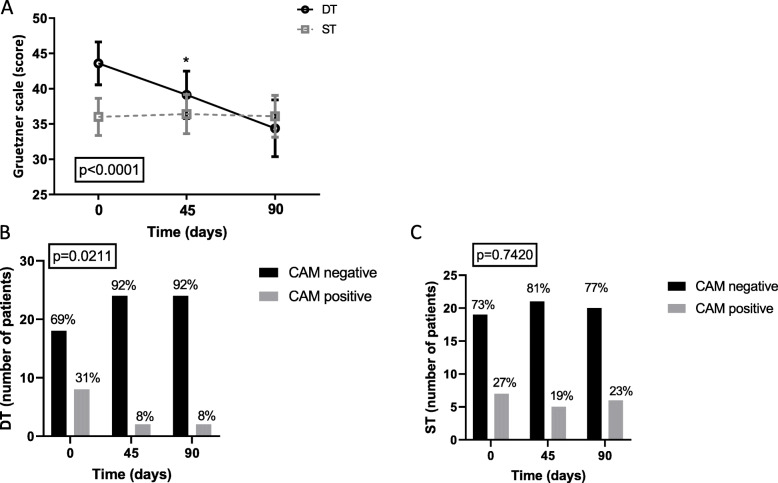
Doll therapy is effective in reducing the caregiver burden and the incidence of delirium. Effect of DT versus ST on the caregiver burden measured with the Greutzner scale (panel **A**) the results of two-way ANOVA for repeated measurements are shown in the box in panel A, significant differences versus baseline are indicated by the star (*). Incidence of delirium in DT group (panels **B**) and in ST group (panel **C**), both absolute number and percentage of patients is shown. Results for the χ2 test for trends are shown in the box

Moreover, we observed a significant reduction of the professional caregiver burden (Fig. 4A).

### DT is effective in reducing incidence of delirium

As secondary outcome, we measured the incidence of delirium with the CAM scale; our hypothesis was that the doll, as transitional object, might catalyze patients’ attention and reduce the risk of delirium. Interestingly in the subjects treated with DT, incidence of delirium was significantly reduced (Fig. 4B).

## Discussion

The use of doll treatment may be useful as a non-pharmacologic approach to control BPSD in patients with moderate to advanced forms of dementia [15]. Despite some evidences on the efficacy of this approach [17], its use is still not widespread because of some ethical concerns and lack of solid scientific evidences. Amongst the ethical concerns feelings of infantilizing the patient [43] and difficulties in finding the target patient for the treatment [44] have been raised.

In our study, after adequate information, no family nor professional caregiver rise objection and refuse the use of DT. We proposed the use of DT only after a careful observation of patients interaction with the doll, this observation allow us to exclude patients that have a neutral or negative interaction with the doll, thus maximizing the possible positive effects of the treatment and avoiding discomfort for the patients. We observed the interaction of the subjects with the doll for 7 days, during this period, only six subjects have a neutral or negative interaction, and none of them becomes aggressive and agitated after DT administration, they simply refuse the doll. We did not find significant differences between residents refusing and accepting the doll, hence we recommend proposing the doll and observing the patients reaction in order to choose the best candidates for the DT.

The lack of solid scientific evidences on DT is mainly due to the difficult in standardization of non-pharmacologic intervention; some attempt towards standardization of DT have been done with well-designed randomized controlled clinical trials [20, 45–47]. The randomized-controlled clinical trials agree on the efficacy of DT in ameliorating BPSD in nursing home residents. In particular Ylmaz & Asiret show a reduction in the agitation and others BPSD and an increase in the patients’ cognitive performance [47]. Here we show that DT was even more effective than ST in reducing agitation and aggressiveness. In our recent work on an acute care geriatric unit we observed the same effect over a shorter period [30].

Beside the effect on agitation and aggressiveness, we observed a reduction of dysphoria and wandering and an amelioration of apathy. The reduction of apathy and dysphoria observed in our study confirms the results of previous non randomized-controlled studies suggesting that DT may stimulate patients’ perception [28], ameliorating their communication abilities, their self-esteem and overall quality of life [48, 49]. Our data suggest that DT is also effective in controlling agitation and/or aggressiveness PRN, nevertheless, we do not find significant difference in chronic antipsychotic drugs during DT. The evaluation of change in chronic antipsychotic treatment was not stated as end point in our study, hence it is not possible to drawn significant conclusion from this observation.

Thanks to the effects on BPSD, DT significantly reduced the perceived professional caregiver burden; this might contribute to higher quality of care for persons living with dementia [50], hence this is one of the main goals of treating BPSD.

Delirium is common amongst older patients and the risk of developing delirium is increased by the presence of cognitive impairment [51, 52]. The prevention of delirium is of paramount importance, ameliorates patients clinical outcome and comfort [53, 54]. Non-pharmacologic approaches have been suggested for the prevention of delirium [53] and, although sparse, data currently available suggest efficacy for some tools as appropriate lighting, the use of calendar and clocks to reduce delirium incidence. Moreover, music therapy have been evaluated as possible tool to reduce delirium, a paper from Jonson and colleagues suggests that music therapy may reduce physiologic variations associated to delirium as hearth rate and systolic blood pressure, however it provides no direct evidence of delirium prevention [55].

This is the first study investigating the possible role of DT in the prevention of delirium in patients affected by dementia. Here we show that patients treated with DT have a lower incidence of delirium measured by the CAM scale. The exact mechanisms is currently unknown, we hypothesize that DT may act on one of the factors causing delirium, according with the Hospital Elder Life Program this are: orientation, therapeutic activities, early mobilization, vision/hearing optimization, oral volume repletion, and sleep enhancement [56]. Interaction with the doll may be considered as a therapeutic activity catalysing patients’ attention and reducing the risk of delirium.

As major limitation of our study, we acknowledged the interaction between the health care professionals and the patients at the moment of DT administration, we cannot exclude an effect of such interaction in calming down the patients. However also in the ST group the staff interact with the patient when administering the treatment, usually the health care professional explain to the patients the treatment that is about to receive and help him/her in taking the drugs, nevertheless this type of behavioral intervention was not standardized. On this regard, it is interesting to underline that the comparison of DT with common clinical practice allow us to generalize our findings.

We gave detailed information on potential benefit of the DT in subjects living with dementia to the nursing home staff, mainly to avoid the concerns about possible infantilization of the patients, however these information may led to detection bias. This limitation is intrinsic to the use of non-pharmacological intervention and to the use of scales that require an evaluation of the patients’ behavior to assess the intervention efficacy.

In conclusion, DT may be an option for the treatment of BPSD in nursing home residents affected by severe to moderate dementia providing a careful evaluation of doll acceptance both from patients and family. On this regard the professional caregiver must be formed in order to correctly present the treatment to the patient and his/her family.

Here we show, for the first time in literature, a possible effect of DT in reducing the incidence of delirium, these data needs to be further explore with an ad hoc designed trial.

## Data Availability

The datasets generated during and/or analyzed during the current study are available from the corresponding author on reasonable request.
